# On the Nature and Mode of Origin of the Lead-Line in the Gums

**Published:** 1881-12

**Authors:** C. Hilton Fagge


### SELECTIONS.

### ON THE NATURE AND MODE OF ORIGIN OF THE LEAD-LINE IN THE GUMS.

More than thirty-four years ago, Dr. Henry Burton, in a communication to the Royal Medical and Chirurgical Society, pointed out that in persons under the influence of lead a peculiar b'ue line could be discovered on the borders of the gums. Mr. Tomes subsequently gave a tolerably complete explanation of its production. But I am not aware that the microscopical appearance of the gingival structures when affected by the metal has hitherto been demonstrated. And having recently had opportunities in two cases of investigating after death the nature of the change, I think it may be interesting to the society to have my observations brought under their notice, particularly as I believe that they afford grounds for defining more accurately than has hitherto been possible the conditions under which the lead-line is developed.

Another circumstance which makes me desirous of drawing the attention of the profession to the subject is the fact that some of the most recent writers on medicine give descriptions of the affection of the gums caused by lead, which seem to me inaccurate and misleading. Thus an eminent physician, lately deceased, spoke in his lectures (published in the *Lancet* in 1872) of "a bluish-purple line next the teeth, and encroaching on them;" and Dr. Aitken, in his well-known text book, says that the metal produces ulceration of the gums and alveolar processes, accompanied by a peculiar blue line. . . along the free margin of the gums. And further on, he remarks that "Many pathologists are inclined to believe that the line is owing to the presence of lead in some peculiar state of combination, as with some of the constituents of the tartar of the teeth." Dr. Burton, however, described the line as on the gums themselves; and he expressly stated that "there was no invariable tumefaction, softening, or tenderness about them," while in another place he seems to have implied that when the gums were ulcerated, tumid, or detached from the teeth by incrustations, such conditions were due to neglect rather than to the metal.

One character of the lead-line, which I have never seen mentioned in print, was some years ago pointed out to me by Sir W. Gull; namely, that it is not continuous, but consists of a series of dots arranged side by side at pretty uniform distances. He used to say that the dots corresponded with vascular points which exist in the normal mucous membrane; and any one who will look at the gums of a few healthy individuals will find that they often present a row of reddish spots, which are precisely similar in position and in size.

The method which I have adopted in examining the lead-line after death, has been to slice off a thin layer of the tissue of the gum at the margin next the teeth, and place it beneath the microscope, using a rather low objective. It is then seen that the discoloration is not uniform, but is distributed in the form of rounded loops, some of which look like minute papillary processes of the deeper layer of the mucous membrane, while others seem to be portions of blood-vessels. Under a higher power the pigmentation is seen to be caused by the presence of granules, which may be scattered at a little distance from one another, or collected into dense masses. I have satisfied myself that these are sometimes situated in the interior of the smallest blood-vessels, and sometimes outside them in the tissue immediately adjacent to their walls.

When the lead-line is well marked it may regularly surround the bases of all the teeth. But it is, perhaps, more commonly imperfect in its development, and then there may be merely two or three dots here and there; generally, these are to be seen in the little processes of gum, which project between the teeth, rather than in the notches which correspond with their centres. On the other hand it sometimes happens that the discoloration is present in what may be termed an exaggerated degree. The margins of the gums may then be uniformly blackened, no separate dots being discoverable; and the staining may extend over the mucous membrane covering the whole of the alveolar process, and even show itself upon that which lines the lips and the interior of the cheeks. In the summer of 1875 a patient was in Guy's Hospital under my care, in whom the inner surface of the lower lip presented a black zone of considerable depth. Some observers have thought that the teeth themselves may become blackened from the same cause; and Mr. Tomes speaks of the tartar as being similarly discolored, especially where it is in contact with the gum. But I hardly think that this can be the case; and I should be disposed to regard any such appearances as accidental, since they are by no means uncommon in persons who cannot be supposed to be under the influence of the metal.

However this may be, it is clear that the so-called blue line is itself really *in* the gum and not *on* its surface. And the blue color seems to me only apparent. When examined with a lens during life, the dots which make up the line are seen to be black. My friend, Dr. F. Taylor, has suggested to me that the reason why as a whole it looks blue is that much of the pigment is seen through a comparatively thick layer of translucent tissue. There are many other examples of black pigments which seem to be blue when imbedded in or beneath similar fibrous textures.

Mr. Tomes some years ago suggested that the coloring material in the blue line on the gums was probably a sulphuret of lead, and he pointed out that the tartar about the teeth is so porous as "readily to admit into its substance fluids charged with animal matter, which may there be decomposed and furnish sulphureted hydrogen." We are now able, I think, to form a still more accurate conception of the mode of production of the line that seems to have suggested itself to Mr. Tomes. We see that the sulphureted hydrogen must diffuse itself into the gingival tissues, and combine with the lead as it is actually circulating in the blood which passes through the vessels of the gum, or as it is contained in the plasma which oozes out of the vessels for the nourishment of the epithelial and other structures. If the lead were previously in combination with the elements of the connective tissue of the gum, the line would not to the naked eye appear dotted; nor would it present the loops and serpentine coils which are seen under the microscope.*

It appears to me probable that the sulphureted hydrogen is often derived directly from portions of meat or of other animal food, which may have become wedged in the angles between the teeth, and undergo decomposition there. For, as I have already remarked, when the line is imperfect the dots which represent its rudimentary condition are often limited to the processes of gum which project upwards into those angles.

---

* My observations are, of course, incompatible with the view advanced by Naunyn in von Ziemssen's "Handbuch" (vol. xv, p. 263), that the lead probably enters the oral cavity from without in the form of particles of the metal. (Oct., 1876).

Mr. Tomes offered, as an alternative, the suggestion that the sulphocyanogen of the saliva might possibly furnish the sulphur for the production of the line. But I think that this is negatived by the limitation of the line to the margin of the gum close to the teeth, and by the fact (which Mr. Tomes pointed out) that wherever there is a gap in the row of teeth the line is wanting. The influence of the saliva would be more equally distributed over all parts of the gums.

It appears, therefore that the lead line may be regarded as a *precipitate* of the metal from the blood, or even, in some sense, as an *excretion.* This view affords an explantion of several points in regard to the line which have hitherto appeared of doubtful interpretation. One is the fact, noticed by Mr. Tomes, that it is sometimes seen in the gums of persons who are not known to have been exposed to the influence of the poison, and who have had none of its symptoms. Mr. Tomes himself suspected that other metals besides lead might sometimes give rise to a similar discoloration. The late Dr. Brinton* considered that in some patients who had taken bismuth he had seen an affection of this kind. But it appears evident from his brief description that he was referring to the reddish-purple border which is commonly present when the gums are in an unhealthy state, and which is altogether different, being simply due to vascular injection. If any other metal were really capable of producing a line resembling the lead line, I think that the fact must before this have become fully recognized. It seems to me probable that whenever the appearance in question is observed, even though the person may present no other indication of having been affected by the poison, it is really caused by lead, but that this metal has in such cases been taken into the body in quantities too small to give rise to symptoms.

The black line being a precipitate of sulphide of lead from the circulating fluid, one can see how it might be produced in course of time by the action of sulphureted hydrogen on the blood constantly flowing through the gingival vessels, although the amount of the metal present in the body at any one moment might be almost infinitesimally minute, and even although the total amount present during all the time in which the line was in process of formation, might be quite inconsiderable.

It is, indeed, a difficulty which stands in the way of the acceptance

* "Brinton on Ulcer of the Stomach," 1857, p. 118.

of this hypothesis: that the lead-line in the gums seems not to be permanent, at least in many instances. Dr. Burton mentions that in a few of his cases it had actually disappeared before the patients left the hospital. Probably it may pass off, if the absorption of the metal ceases, even though the internal structures may still retain the deposits of lead, which are known to occur in those who have been exposed to its influence. I do not know in what other way one can explain the fact that the administration of the iodide of potassium to a person who has been poisoned by lead, but who is placed under conditions which no longer involve its entrance into the body, is sometimes followed by the development of a line on the gums which had previously been wanting. I have observed this on three occasions at least, and Dr. Frank Smith, of Sheffield, has independently noticed it. It is strictly parallel to the well-known circumstance that when iodide of potassium is given to a patient affected with chronic plumbism, but withdrawn from the direct influence of the poison, the urine begins to contain lead, although none could before be discovered in it. The only difficulty is to understand how the lead-line came to be absent until the iodide began to be administered. But this difficulty is removed by the supposition that it really had existed at one time, but had disappeared during the interval that had elapsed since the metal ceased to be absorbed.

Another conclusion, which is deducible from the view suggested by Mr. Tomes with regard to the line in the gums, is that in a person who keeps his teeth clean it may be wanting even though he may be really suffering from lead poisoning. I have long made it a rule to attach no importance to the absence of the line in individuals with very clean teeth and gums; but I cannot cite any case in which my caution was justified by the result. I have, however, more than once told a patient to leave off cleaning his teeth when he was taking iodide of potassium, in order that the conditions might be favorable for the development of the line.

It ought also to follow from Mr. Tomes' theory that the sulphide of lead should be deposited in all other parts of the body which are exposed to the influence of sulphureted hydrogen. This gas is often present in the intestine, the mucous surface of which ought accordingly to be blackened, at least as much as the gums. I am not aware that this has hitherto been noticed; but it is possible that the frequency with which the intestinal lining membrane is found discolored under other circumstances may have prevented observers from attaching any importance to such an appearance, even if it should have been present. In November, 1875, I made an autopsy in the case of a man, æt. 43, a painter, who had had colic fifteen years back, and who, while in the hospital had complained of pain about the splenic flexure of the colon. I found in this part of the bowel, as well as in the transverse colon, some scattered cicatrices, with puckering of the mucous membrane. They were slate-colored, but I did not at the time notice that their appearance differed from that of the scars which are found after ulceration from dysentery or other causes.—*C. Hilton Fagge in Journ. Brit. Dental Association.*

